# COVID-19 Vaccination and Pulmonary Nodules: A Narrative Review of Causality, Detection Bias and Thoracic Imaging Pitfalls

**DOI:** 10.3390/vaccines14090731

**Published:** 2026-08-25

**Authors:** Jiqiu Hou, Yiwen Li, Meimei Tao

**Affiliations:** 1 Department of General Internal Medicine, Dongfang Hospital, Beijing University of Chinese Medicine, Beijing 100078, China; jiqiu.hou@liverpool.ac.uk; 2 Department of Pharmacology and Therapeutics, Institute of Systems, Molecular and Integrative Biology, Faculty of Health and Life Sciences, University of Liverpool, Liverpool L69 7ZX, UK; 3 Department of Pharmacy, Emergency General Hospital, Beijing 100028, China; 4 Department of Pulmonary and Critical Care Medicine, Beijing Anzhen Hospital, Capital Medical University, Beijing 100029, China

**Keywords:** COVID-19 vaccination, pulmonary nodules, lung nodule, vaccine safety, [18F]FDG-PET/CT, lymphadenopathy, post-COVID lung abnormalities, artificial intelligence, lung cancer screening

## Abstract

**Background/Objectives**: Concern that COVID-19 vaccination causes pulmonary nodules persists because vaccination coincided with expanded computed tomography (CT), low-dose CT (LDCT) screening, post-COVID imaging and artificial intelligence (AI)-assisted detection. This review asks whether the current literature supports causality and how vaccination history should inform thoracic imaging. **Methods**: A focused search of PubMed, PubMed Central and the Cochrane Library was performed through 15 July 2026. Evidence was classified as direct or contextual; no PRISMA screening, risk-of-bias scoring or quantitative synthesis was performed. **Results**: Direct evidence remains sparse. One case report was too confounded for inference. A two-sample Mendelian randomization study found no broad lung disease risk signal, but nodules were not modeled and the heterogeneous endpoints were exploratory. An ecological study of 1,616,750 samples linked rising detection to SARS-CoV-2 infection waves and AI-assisted reading; lacking individual vaccination data, it cannot establish whether vaccination affected detection. Screening interruption produced the opposite pattern: Lung-RADS 4 nodules rose from 8% to 29%. The most reproducible post-vaccination thoracic finding is regional lymph-node activation on [18F]fluorodeoxyglucose positron emission tomography/computed tomography ([18F]FDG-PET/CT), an expected immune response rather than a parenchymal nodule. **Conclusions**: Current evidence is insufficient to establish vaccination as an independent, population-level cause of pulmonary nodules. Vaccination history should guide [18F]FDG-PET/CT interpretation; CT-detected parenchymal nodules warrant standard risk stratification.

## 1. Introduction

COVID-19 vaccination has reduced severe disease and death on a population scale, yet questions about vaccine safety continue to shape public confidence. One recurring concern is whether a pulmonary nodule discovered after vaccination represents vaccine-induced lung injury. The concern is understandable. Pulmonary nodules are common on CT-based screening and incidental imaging, and their apparent frequency depends strongly on the population screened, the CT protocol, the nodule-size threshold and the reporting workflow. In the National Lung Screening Trial, 24.2% of LDCT screening tests were positive across three rounds [1], while NELSON-era analyses and Fleischner Society guidance emphasize that small non-calcified nodules are frequent and call for risk-stratified management rather than exposure-driven attribution [2,3]. Against that background, some temporal overlap between vaccination and nodule detection is almost inevitable. Attributing a nodule to the vaccine without adequate evidence, however, can delay risk stratification, heighten patient anxiety and reinforce vaccine hesitancy.

Establishing causation here is methodologically demanding because vaccination coincided with several concurrent secular trends, including widespread SARS-CoV-2 infection, expanded chest CT and LDCT screening, thin-section reconstruction and AI-assisted interpretation. Each of these trends independently affects how many nodules are detected. A nodule identified after vaccination may be genuinely incident, or pre-existing but newly visualized, newly reported under changed thresholds, or newly acted upon; these possibilities carry different mechanisms, and only the first implies new disease. Separating them requires distinguishing a true rise in incidence from increased ascertainment [4], which temporal order alone cannot achieve. In routine practice, there is usually no pre-vaccination baseline scan and, hence, no counterfactual against which a newly observed nodule can be judged. In the Bradford Hill framework, temporality is only one causal consideration [5]; in this setting, it is further confounded by infection-related sequelae, greater imaging intensity, shifting reader thresholds and algorithmic detection.

A second source of confusion is anatomical. The thoracic finding most reproducibly associated with COVID-19 vaccination is not a pulmonary lesion but reactive regional lymphadenopathy: transient hypermetabolic activation of the nodes draining the injection site, most often the ipsilateral axillary and, less frequently, supraclavicular nodes, demonstrable as increased uptake on [18F]fluorodeoxyglucose positron emission tomography/computed tomography ([18F]FDG-PET/CT) and as nodal enlargement on CT or ultrasound [6,7]. This represents an expected immune response rather than a pathological lung finding, yet it can be misinterpreted as malignant nodal disease during cancer staging or surveillance. It is distinct, both anatomically and mechanistically, from a pulmonary parenchymal nodule, which occupies a different tissue compartment and carries a substantially broader differential diagnosis. A vaccine-focused review, therefore, has to separate this well-characterized, self-limited nodal phenomenon from the proposition that vaccination generates pulmonary parenchymal nodules, for which the current evidence remains insufficient to establish causation.

Accordingly, this narrative review appraises the current evidence on COVID-19 vaccination and pulmonary nodules, organizing the literature by evidentiary role rather than by publication type alone: direct evidence on vaccination and nodules or lung disease risk, contextual evidence on imaging pitfalls, and clinical guidance for nodules detected after vaccination. It asks two linked questions: whether the literature supports a causal claim, and how vaccination history should be used in thoracic imaging without conflating nodal immune activation with parenchymal disease. The aim is not to dismiss patient concern but to place it within a defensible causal and imaging framework.

## 2. Materials and Methods

This study is a narrative evidence review rather than a formal systematic review or meta-analysis. The final search date was 15 July 2026. A focused search of PubMed, PubMed Central and the Cochrane Library (Cochrane Database of Systematic Reviews and CENTRAL; no date restriction applied) was performed. Core search strings combined COVID-19 vaccination terms (COVID-19 vaccin*, SARS-CoV-2 vaccin*, mRNA vaccin*, booster) with pulmonary nodule terms (pulmonary nodule*, lung nodule*, micronodule*, nodule detection, nodule progression), lung disease outcomes (lung cancer, pneumonia, idiopathic pulmonary fibrosis, pulmonary embolism, pneumothorax, sarcoidosis, asthma, COPD), imaging terms ([18F]FDG-PET/CT, lymphadenopathy, axillary node, post-COVID CT) and detection bias terms (artificial intelligence, AI-assisted detection, low-dose CT, screening). The Cochrane Library search identified no systematic reviews or protocols addressing COVID-19 vaccination in relation to pulmonary nodules; a CENTRAL search retrieved nine trial records (seven independent trials) concerning vaccine safety, immunogenicity or post-vaccination lymphatic and imaging endpoints, none of which evaluated COVID-19 vaccination as a cause of pulmonary parenchymal nodules. Title/abstract and full-text screening was performed independently by two authors (J.H. and Y.L.); disagreements were resolved by discussion and, where necessary, adjudication by the senior author (M.T.). The exact final PubMed/PubMed Central query strings and the Cochrane Library/CENTRAL search syntax, together with the numbers of records retrieved before and after de-duplication, are reported in the Search Strategy Appendix (Supplementary Materials).

Records were eligible when they addressed COVID-19 vaccination together with pulmonary nodules, lung disease outcomes, thoracic imaging findings, post-COVID CT abnormalities, AI-assisted nodule detection or pulmonary nodule management. Peer-reviewed studies with available abstracts or full texts were prioritized. Case reports were retained only when they addressed vaccination together with nodular or granulomatous lung findings. Public commentaries, question-and-answer pages, unverifiable secondary materials and conference-only claims without verifiable full text were excluded from the evidence table; abstract-only materials were screened for awareness but were not used as substantive evidence unless the bibliographic details and the underlying report could be independently verified.

Evidence was then classified as direct or contextual. Direct evidence comprised studies that assessed COVID-19 vaccination in relation to pulmonary nodules, nodule detection, nodule progression or lung disease outcomes. Contextual evidence comprised imaging studies of vaccine-associated lymphadenopathy, case reports of rare immune-mediated disease, post-COVID CT follow-up studies, AI-assisted detection studies and pulmonary nodule guidelines. This classification was prespecified because studies of imaging pitfalls can inform differential diagnosis yet cannot, by themselves, establish nodule causation. Because this was a narrative review, no PRISMA flow diagram, formal risk-of-bias scoring, pooled estimate or certainty-of-evidence grading was generated; given the heterogeneity in design, exposure and outcome definitions, no pooled estimate was attempted. The search should, therefore, be read as a focused search of selected biomedical databases and publisher platforms, not an exhaustive search of Embase, Web of Science or all grey-literature sources. The synthesis instead emphasizes the direction and strength of the evidence, the specificity of the imaging finding and the plausibility of competing explanations. This qualitative approach is appropriate for mapping the causal question and the clinical interpretation problem, but it cannot estimate incidence, prevalence or rare individual risk. AI-assisted tools:Codex (OpenAI ChatGPT, version 26.819.11345) and Claude Opus 4.8 (Anthropic), were used only for language refinement and formatting (see the Use of Artificial Intelligence statement).

## 3. Evidence Synthesis

The evidence base is best read as a hierarchy. Case reports can flag temporally associated events but cannot separate causation from coincidence. Genetic causal inference and large real-world datasets carry more weight, though they too have endpoint and measurement limits. Studies of lymphadenopathy and post-COVID lung change are contextual: they illuminate plausible misclassification pathways and competing explanations, but they are not direct proof of vaccine-induced pulmonary nodules. Accordingly, the evidence is organized into three tiers: direct (individual-level vaccination exposure with pulmonary nodule outcomes), indirect (vaccination with broader lung disease outcomes or ecological detection trends), and contextual (imaging pitfalls, AI-assisted detection, post-COVID abnormalities and guidelines).

### 3.1. Direct Evidence on Vaccination, Pulmonary Nodules and Lung Disease Risk

Direct evidence is limited and uneven. The most explicit signal came from a single CARE-compliant case report: a postpartum woman with a few (3–5) randomly distributed 1–3 mm pulmonary micronodules and benign thyroid cystic nodules after four pre-pregnancy mRNA vaccine doses [8]. The report is clinically useful in documenting exactly the temporal sequence that worries patients and clinicians, and the authors proposed a speculative multifactorial explanation linking vaccine history, lifestyle change, anxiety and metabolic factors; the nodules were managed conservatively and neither progressed nor regressed during follow-up. Its inferential value is nonetheless low, because pregnancy, postpartum immune adaptation, psychological stress, disturbed sleep, reduced activity and dietary change all offer competing explanations.

Higher-level evidence does not support a broad lung disease risk signal, although it does not test pulmonary nodules directly. A two-sample Mendelian randomization study used seven genetic instruments (indirect evidence, since it evaluated broad lung disease outcomes rather than pulmonary nodules) meeting genome-wide significance (F-statistics ranged from 29.8 to 38.4, above the weak-instrument threshold) to test COVID-19 vaccination status against 14 lung disease outcomes, with inverse-variance weighting as the primary method and MR-Egger regression, weighted-median estimation and Bonferroni and Benjamini–Hochberg corrections as sensitivity and multiplicity checks [9]. It reported no association with overall lung cancer (*p* = 0.78), pneumonia (*p* = 0.282), idiopathic pulmonary fibrosis (*p* = 0.486), pulmonary embolism (*p* = 0.267), pneumothorax (*p* = 0.73), sarcoidosis (*p* = 0.732), asthma or several other outcomes. The original report, however, also noted inverse-variance-weighted heterogeneity for overall lung cancer, COPD and forced vital capacity (FVC), so those three endpoints are treated here as exploratory rather than definitive. Endpoint labels and some *p*-value attributions are, moreover, not fully aligned across its abstract, methods and results sections. Several further features limit extrapolation: the exposure data came from the Finnish FinRegistry; analyses were confined to European-ancestry cohorts; genetically predicted vaccination status may partly capture behavioral and health service factors; and pulmonary nodules were not modeled as a discrete imaging endpoint. For this review, this study is, therefore, read as a broad null signal for major lung disease outcomes rather than as endpoint-specific proof about pulmonary nodules. Notably, the original authors themselves acknowledged that their exposure genome-wide association study could not distinguish between vaccine platforms and called for platform-specific genetic data, which reinforces the concern that a class-wide null could obscure a platform-specific signal, median 33.9.

The evidence closest to public concern comes from detection-trend data, but it remains ecological rather than individual-level causal evidence. A multicenter cross-sectional study analyzed 1,616,750 clinical samples (1,102,605 outpatient and 514,145 health examination) from 23 Chinese centers of varying tiers between 2019 and 2023 [10]. Nodule detection rose in two waves (2020 to 2021 and 2023), with a plateau in 2021 to 2022, and increased more steeply among outpatients, among men and at university-affiliated or provincial hospitals. This study observed that the period when national vaccination coverage approached 90% overlapped with a detection plateau, whereas detection tracked SARS-CoV-2 infection waves and the introduction of AI-assisted reading. That temporal pattern should be labeled an ecological observation, not evidence that vaccination reduced, prevented or failed to affect individual nodule detection: the study contained no individual-level vaccination histories, infection timing or pre-vaccination baseline CT, which precludes causal inference between personal vaccination status and nodule detection. Notably, the proportion of CT-suspected lung tumors or cancers stayed low and stable and did not track the nodule curve. A separate multicenter CT screening cohort of 18,906 examinations found higher detection of solid nodules and fibrotic-like abnormalities after the epidemic period: 11,513 nodules in all, of which 841 were high-risk, yet the high-risk proportion did not rise, and vaccination exposure was not directly assessed [11]. For screening practice, the shared message is that a higher detection rate does not equate to a greater burden of high-risk nodules or lung cancer. This ecological pattern is, therefore, limited to detection trends and ascertainment effects and must not be read as arguing for or against vaccine causality.

A countervailing example comes from screening interruption rather than screening expansion. In a US lung cancer screening program, Van Haren et al. reported that COVID-19-related LDCT suspension and reduced attendance were followed by a rise in Lung-RADS 4 nodules suspicious for malignancy, from 8% before the disruption to 29% after screening resumed (*p* < 0.01) [12]. This points in the opposite operational direction from studies showing more small-nodule detection under expanded imaging or AI-assisted sensitivity. The two patterns are reconcilable: Van Haren et al. [12]. captured delayed screening and delayed diagnostic entry, whereas Li et al. [10] and He et al. [11]. mainly captured infection-era imaging intensity, hospital tier, screening expansion and AI-assisted sensitivity. Read together, they strengthen the core inference that observed nodule patterns depend heavily on the screening chain and on ascertainment conditions rather than on vaccination timing.

### 3.2. Vaccine-Associated Imaging Findings Are Mainly Nodal, Not Parenchymal

The clearest vaccine-related thoracic signal is reactive lymphadenopathy. Reviews and oncologic imaging series describe a recurring pattern of ipsilateral axillary or regional nodal uptake on [18F]FDG-PET/CT after COVID-19 vaccination [6,7]. The quantitative estimates most often cited in this review come from one representative single-center oncologic [18F]FDG-PET/CT series of 140 patients: 75 (54%) showed FDG-avid lymph nodes ipsilateral to the injection, with a mean SUVmax of 5.1; uptake was more frequent after Moderna than after Pfizer-BioNTech (72% vs. 43%), and, in a subset, the nodes would have been read as suspicious for metastasis had the vaccination history been unknown [6]. These figures should, therefore, be read as illustrative single-center estimates, not as pooled incidence values. Comparable ipsilateral axillary lymphadenopathy has also been documented after inactivated COVID-19 vaccines on ultrasonography, indicating that this reactive nodal response is not confined to mRNA platforms [13]. The finding is directly relevant to patients with breast cancer, melanoma, lymphoma or other malignancies in which nodal status drives management. Beyond nodal uptake, isolated reports describe increased [18F]FDG avidity, prompting evaluation of a pulmonary nodule after vaccination [14] and axillary adenopathy detected on staging imaging after vaccination [15]; these too represent imaging pitfalls, in which recent vaccination, rather than a new parenchymal lesion, accounts for the finding.

This evidence should not be extrapolated to pulmonary parenchymal nodules. Nodal activation reflects an expected immune response at regional drainage sites, whereas a pulmonary nodule is a parenchymal lesion with a broad differential diagnosis. The distinction matters because [18F]FDG-PET/CT nodal uptake and CT-detected lung nodules lead to different clinical questions, different follow-up strategies and different causal inferences. Conflating the two invites errors in both directions: over-attributing CT nodules to the vaccine and under-recognizing vaccine-associated nodal uptake that can distort [18F]FDG-PET/CT interpretation.

### 3.3. Rare Immune-Mediated Case Reports and Differential Diagnosis

A handful of case reports describe sarcoidosis-like disease or granulomatosis with polyangiitis after COVID-19 vaccination [16,17]. Because granulomatous and vasculitic diseases can produce pulmonary nodules or nodule-like lesions, these reports matter for clinical vigilance; their value lies in differential diagnosis, not in estimating population-level risk. Within this group, the two reports differ in a way that matters for classification: the granulomatosis-with-polyangiitis case presented with multiple pulmonary nodules that regressed under immunosuppression, indicating an inflammatory rather than neoplastic parenchymal process [16], whereas the mRNA vaccine-associated uveitis case led to a diagnosis of sarcoidosis, in which the thoracic finding was right-lung ground-glass/granular opacity with hilar and mediastinal lymphadenopathy—nodal and parenchymal ground-glass change rather than a discrete pulmonary nodule—confirmed by parotid-gland biopsy [17]. Such reports, therefore, inform the differential diagnosis rather than parenchymal nodule causation.

A further cautionary example is a woman who developed diffuse bilateral pulmonary nodules and ground-glass opacities after ChAdOx1-S vaccination and was ultimately diagnosed, on biopsy, with pulmonary talcosis attributable to years of cosmetic talc inhalation; the vaccination was only the temporal trigger that prompted evaluation, not the cause of the nodules [18]. This illustrates that nodules discovered after vaccination require a complete differential diagnosis and that a temporal association can readily mislead when a pre-existing or exposure-related condition is present. Because the cited studies span mRNA, inactivated and ChAdOx1-S platforms, which differ in formulation and immune response, any class-wide null conclusion could obscure a platform-specific signal; the vaccine platform is, therefore, identified per the study in Table 1, and this heterogeneity is treated as a limitation.

The practical implication is conditional. When pulmonary nodules occur alongside persistent fever, uveitis, rash, bilateral hilar or mediastinal lymphadenopathy, sinonasal symptoms, hemoptysis, renal involvement or abnormal autoimmune markers, the differential should be broadened. When nodules are isolated incidental CT findings, the same case reports should not be read as evidence that vaccination caused them. The main clinical characteristics of the individual case reports of parenchymal nodular findings are summarized in Table S1 (Supplementary Materials).

### 3.4. Post-COVID Lung Abnormalities and Screening-Era Detection

For parenchymal lung change, SARS-CoV-2 infection is a more biologically proximal explanation than vaccination. A systematic review and meta-analysis of one-year CT findings after COVID-19 reported residual abnormalities—ground-glass opacity, consolidation, nodules or masses, parenchymal bands and reticulation [19]—and a three-year longitudinal cohort found persistent radiological abnormalities in a subset of hospitalized survivors [20].

This infection-related pathway matters because nodules are interpreted in real clinical workflows, not in an exposure-isolated setting. Early lung cancer and COVID-19 can both present with ground-glass opacity [21], so post-infectious change, pre-existing small nodules and screening-detected lesions may coexist in the same follow-up population.

Screening behavior and technology also shifted. More people underwent chest imaging because of infection, persistent symptoms, health examinations or anxiety, while AI-assisted software raised sensitivity for small nodules. Systematic reviews caution that this gain in sensitivity can come with lower specificity and more false-positive surveillance [22,23]. Increased detection is, therefore, not the same as increased incidence. Table 2 summarizes the representative evidence, potential pitfalls and clinical implications across these evidence domains, to help avoid misattribution.

## 4. Discussion

### 4.1. Causal Interpretation: What Can and Cannot Be Concluded

Taken together, the published evidence identified in this narrative review is insufficient to establish COVID-19 vaccination as an independent, population-level cause of pulmonary nodules—a conclusion that is cautious rather than dismissive. Direct studies are few, and none has yet combined individual-level vaccination data, pre-vaccination CT, infection history, AI workflow and standardized longitudinal nodule measurement. The available population-level trend data are useful for generating hypotheses, but they can prove neither that vaccination protects against nodule detection nor that vaccination has no individual-level effect. Within those limits, the evidence points more consistently toward infection, screening intensity, screening interruption and detection technology as the main drivers of observed detection patterns.

The strongest public claim is usually temporal: a patient was vaccinated and later had a nodule detected. For causal inference, that is not enough. A credible causal claim would require a consistent excess risk after vaccination, a plausible risk window, a dose- or platform-related pattern, separation from SARS-CoV-2 infection, control for baseline imaging and evidence of incident formation or accelerated growth, none of which the current literature provides. The same standard must be applied symmetrically; if temporal coincidence cannot establish vaccine harm, neither can it establish a protective or detection-slowing effect.

The evidence does support a narrower, clinically useful statement: vaccination history matters for imaging interpretation, especially [18F]FDG-PET/CT, because regional lymphadenopathy can mimic nodal disease. That statement is specific, evidence-based and actionable, and it should not be generalized into a claim that vaccination causes CT-detected pulmonary nodules. Table 3 summarizes, for each causal criterion, the current evidence and the remaining gap.

### 4.2. Biological Plausibility and Anatomical Specificity

Biological plausibility should be judged at the level of the specific imaging finding. A vaccine-induced immune response in draining lymph nodes is plausible and repeatedly observed on [18F]FDG-PET/CT. A generalized pathway from intramuscular vaccination to new pulmonary parenchymal nodules, by contrast, has not been demonstrated in population-level imaging data. Treating nodal metabolic activity as equivalent to parenchymal nodule formation is the conceptual error most worth avoiding.

Rare immune-mediated case reports do not overturn this distinction. Sarcoidosis-like disease and ANCA-associated vasculitis can involve the lung, and vaccination may be temporally associated with immune activation in susceptible individuals. Such reports justify diagnostic awareness when systemic features are present; they do not show that routine incidental nodules after vaccination share the same mechanism.

### 4.3. A Practical Imaging and Clinical Interpretation Framework

For clinicians and radiologists, the task is to prevent misattribution in both directions. Vaccine history should be recorded, because it can explain nodal findings and guide [18F]FDG-PET/CT interpretation; at the same time, pulmonary nodules should be assessed by established principles—size, density, morphology, multiplicity, growth, smoking history, age, cancer history, family history and guideline-based follow-up [3,24,25,26,27].

A useful first question is whether the finding is nodal or parenchymal. Recent ipsilateral axillary uptake after vaccination usually supports a reactive reading, whereas parenchymal nodules require standard CT-based risk assessment. Systemic symptoms or autoimmune features should prompt a broader differential, including granulomatous disease or vasculitis. Table 4 summarizes practical scenarios for interpreting pulmonary or thoracic findings detected after COVID-19 vaccination, together with the more plausible explanation and recommended interpretation for each.

### 4.4. Implications for Vaccine Safety Communication

Vaccine safety communication should avoid two extremes. It should not dismiss patient concern merely because the evidence for causality is weak—discovering a nodule soon after vaccination can cause real anxiety—nor should it present temporal association as causal proof. A transparent message is more accurate: pulmonary nodules can appear after vaccination because both vaccination and CT imaging are common, but current evidence does not show that vaccination independently causes them.

This framing protects both public trust and clinical safety. Over-attribution to vaccination may discourage immunization and divert attention from lung cancer risk assessment, while under-recognition of vaccine-associated lymphadenopathy may trigger unnecessary procedures or inaccurate staging. A balanced approach keeps documented vaccine-related imaging effects separate from unsupported causal claims about parenchymal nodules.

### 4.5. Research Priorities

Future studies should define both exposure and outcome more precisely. Exposure variables should capture vaccine platform, dose number, booster status, injection site, the interval between vaccination and CT, and prespecified risk windows. Outcome variables should distinguish incident nodules, newly detected pre-existing nodules, nodule growth, density change, high-risk nodules and lung cancer.

The most informative designs would be prospective multicenter cohorts, nested case–control studies, target-trial emulations or self-controlled case series with prespecified short risk windows. These should control for SARS-CoV-2 infection history and severity, baseline CT availability, smoking and occupational exposures, CT acquisition parameters, AI software version and reader workflow. Studies of nodule progression should use standardized volumetric or density measurement to separate true growth from technical variation.

### 4.6. Limitations of This Review

This review is narrative rather than systematic, so it does not include PRISMA-style screening, formal risk-of-bias assessment, certainty-of-evidence grading or quantitative synthesis. The search strategy was designed to map a focused causal and imaging interpretation question rather than to estimate incidence or prevalence, and the selected-source approach may have missed studies indexed only in Embase, Web of Science or specialist grey-literature repositories. The direct evidence base is small, and some potentially relevant studies did not report vaccination as an exposure or pulmonary nodules as a separate endpoint; several contextual studies address related imaging phenomena rather than the causal question directly. Importantly, the largest detection-trend study contained no individual-level vaccination histories, so its temporal overlap with vaccination coverage cannot establish individual causality in either direction. Abstract-only claims without verifiable full text were not used as substantive evidence. These constraints bound the strength of the conclusion: the review can state that the published evidence identified here does not support vaccination as an independent cause of pulmonary nodules, but it cannot exclude rare individual immune-mediated events or effects in highly selected subgroups. That distinction should be preserved in both clinical communication and future research.

## 5. Conclusions

The published evidence identified by this narrative review is insufficient to establish COVID-19 vaccination as an independent, population-level cause of pulmonary nodules. The post-pandemic rise in nodule detection is more coherently explained by infection-related lung abnormalities, wider CT and LDCT use, AI-assisted detection, changed screening behavior, screening interruption and baseline patient risk. Ecological timing should not be converted into individual-level causal claims, whether pro- or anti-vaccine. Vaccination history remains important for interpreting regional lymphadenopathy, especially on [18F]FDG-PET/CT, but it should not replace standard evaluation of CT-detected parenchymal nodules. For pulmonary parenchymal nodules, the safest approach is guideline-based risk stratification, longitudinal comparison and attention to the systemic features that would justify a broader differential diagnosis.

## Figures and Tables

**Table 1:** Direct evidence on COVID-19 vaccination, pulmonary nodules and lung disease risk.

Study	Design	Population or Endpoint	*N* Patients/Samples/Examinations	*N* Pulmonary Nodules/Lesions	Main Finding	Interpretation for Causality
Chen et al., 2025 [8]	CARE-compliant case report	One postpartum patient after four pre-pregnancy mRNA doses	1 patient	3–5 micronodules (plus 2 thyroid cystic nodules)	Diffuse 1–3 mm pulmonary micronodules with benign thyroid cystic nodules; managed conservatively, nodules stable on follow-up.	Hypothesis-generating only. Pregnancy, postpartum immune state, anxiety, sleep loss, reduced activity and diet change were major confounders; the proposed multifactorial mechanism remains speculative.
Niu et al., 2025 [9]	Two-sample Mendelian randomization	45,202 vaccinated and 374,178 controls (Finnish FinRegistry); 7 instruments; 14 lung disease outcomes	45,202 vaccinated; 374,178 controls	Not reported (nodules not modelled)	No broad association with major lung disease outcomes; heterogeneity was reported for overall lung cancer, COPD and FVC, and some endpoint/*p*-value labeling differed across manuscript sections. Representative inverse-variance-weighted odds ratios were 0.999 (0.625–1.595) for overall lung cancer, 1.008 (0.874–1.163) for asthma, 0.909 (0.590–1.403) for COPD/bronchiectasis, 1.00 (0.998–1.003) for idiopathic pulmonary fibrosis, 1.309 (0.813–2.106) for pulmonary embolism, 1.108 (0.618–1.984) for pneumothorax and 0.888 (0.449–1.753) for sarcoidosis.	More informative than case reports for broad lung disease outcomes, but exploratory for the heterogeneous endpoints (overall lung cancer, COPD and FVC) and not a direct nodule analysis.
Li et al., 2026 [10]	Multicenter real-world cross-sectional study	1,616,750 samples (1,102,605 outpatient; 514,145 health-exam) from 23 Chinese centers, 2019–2023	1,616,750 samples (1,102,605 outpatient; 514,145 health-exam)	Not reported as a discrete count (ecological rates)	Detection rose in two waves (2020–2021, 2023) with a 2021–2022 plateau; SARS-CoV-2 infection waves and AI-assisted reading were stronger ecological correlates than vaccination coverage; suspected cancer stayed low and stable.	Most relevant population-level detection evidence, but ecological only. No individual vaccination histories; cannot infer personal vaccine-nodule causality in either direction.
He et al., 2025 [11]	Multicenter retrospective CT screening cohort	18,906 chest CT examinations before and after the epidemic period	18,906 CT examinations	11,513 nodules (841 high-risk)	11,513 nodules detected (841 high-risk); solid nodules and fibrotic-like changes increased, but the high-risk proportion did not.	Supports infection- and screening-era effects. Vaccination exposure was not directly assessed.
Van Haren et al., 2021 [12]	Single-program screening cohort, before vs. after disruption	US LDCT screening program interrupted by COVID-19	Not reported (program-level screening cohort)	Not reported as absolute counts (Lung-RADS 4 rose 8%→29%)	Lung-RADS 4 nodules suspicious for malignancy rose from 8% to 29% after screening resumed (*p* < 0.01); no-show rate rose from 15% to 40%.	Opposite operational direction to screening expansion. Illustrates that ascertainment conditions, not vaccination, drive high-risk nodule proportions.

Note: *p*-values, sample sizes and subgroup patterns are taken from the published abstracts or full texts. Mendelian randomization endpoints with significant heterogeneity should be read as exploratory. COPD, chronic obstructive pulmonary disease; FVC, forced vital capacity. The columns for the number of patients/samples and the number of pulmonary nodules or lesions report the values given in each original study; cells marked “not reported” or “not reported as a discrete count” indicate that the study did not provide that figure, and no value has been imputed and no pooling is implied.

**Table 2:** Contextual evidence relevant to misattribution and clinical interpretation.

Evidence Domain	Representative Evidence	Potential Pitfall	Clinical Implication
Regional lymph-node activation after vaccination	Reviews and oncologic series describe ipsilateral axillary or regional nodal uptake on [18F]FDG-PET/CT after vaccination [6,7]. Skawran et al. reported 75/140 (54%), mean SUVmax 5.1 and Moderna vs. Pfizer-BioNTech uptake of 72% vs. 43% [6].	May be mistaken for malignant nodal disease in oncology patients.	Record vaccine date, injection site and dose number before [18F]FDG-PET/CT; interpret nodal uptake in temporal and oncologic context.
Rare granulomatous or autoimmune case reports	Sarcoidosis-like disease and granulomatosis with polyangiitis have been described after vaccination [16,17].	Can include pulmonary nodules, but evidence is limited to rare case reports.	Consider these diagnoses only when nodules accompany systemic, ocular, sinonasal or renal features or autoantibodies.
Post-COVID lung abnormalities	Residual findings after COVID-19 include ground-glass opacity, consolidation, nodules or masses, bands and reticulation [19,20].	Infection is a more biologically proximal cause of parenchymal change than vaccination.	Account for infection history and severity when interpreting new or persistent CT findings.
Overlap with early lung-cancer imaging	COVID-19 and early-stage lung cancer may both present with ground-glass opacity [21].	Misclassification can occur during follow-up, screening or incidental detection.	Use guideline-based follow-up and growth assessment rather than temporal attribution alone.
Screening interruption and risk concentration	After LDCT suspension, Lung-RADS 4 nodules rose from 8% to 29% once screening resumed [12].	Points opposite to screening-expansion data; may be misread as rising disease risk.	High-risk nodule proportions track screening access and continuity; report interruption or expansion context alongside detection figures.
AI-assisted nodule detection	AI may improve sensitivity but can reduce specificity and increase false-positive surveillance [22,23].	Detection rates can rise without a true increase in incident disease.	Report AI use, software version and reader workflow in future epidemiologic studies.

**Table 3:** Causal criteria for a relationship between COVID-19 vaccination and pulmonary nodules: requirements, current evidence and remaining gaps.

Causal Criterion	Current Evidence	Remaining Gap
Consistent excess risk after vaccination (strength and consistency of association)	We found no consistent excess. The two-sample Mendelian randomization study reported no association with major lung disease outcomes [9], and ecological detection-trend data tracked SARS-CoV-2 infection waves and AI-assisted reading rather than vaccination coverage [10]. Only isolated case reports assert a temporal association [8,16,18].	No individual-level study has demonstrated a reproducible excess of incident pulmonary nodules after vaccination relative to an unvaccinated or pre-vaccination comparator.
Plausible, prespecified risk window (temporality)	Reported intervals are highly variable, ranging from approximately 2 months [18] and within about 3 weeks [16] to an approximately 18-month preconception-to-postpartum span [8]. No study applied a prespecified risk window.	A defined biological risk window (for example, through a self-controlled case-series design) has not been tested for parenchymal nodules.
Dose- or platform-related pattern (biological gradient)	The cited studies span mRNA vaccines (four preconception doses [8]; a single dose [16]), an mRNA-predominant [18F]FDG-PET/CT series (Moderna 72% vs. Pfizer-BioNTech 43%) [6], an inactivated vaccine [13] and ChAdOx1-S [18], but none relates nodule outcomes to dose number or platform.	No dose–response or platform-stratified analysis is available for pulmonary parenchymal nodules, so a platform-specific signal can be neither excluded nor confirmed.
Separation from SARS-CoV-2 infection (specificity)	Post-COVID CT studies show residual parenchymal abnormalities attributable to infection, a more biologically proximal cause [19,20], and the largest ecological study lacked individual infection timing [10]. One case reported no SARS-CoV-2 infection history, yet other confounders remained [8].	No study has jointly modelled vaccination and individual infection history to isolate an independent vaccine effect.
Control for baseline imaging and ascertainment	Pre-vaccination baseline CT is rarely available; a normal pre-vaccination chest radiograph was documented in only one case [16], and elsewhere micronodules were an incidental finding on routine postpartum imaging [8]. Detection rose with expanded CT and LDCT use and AI-assisted reading [10,11,22,23], and screening interruption raised high-risk proportions independently of vaccination [12].	No cohort has provided systematic pre- and post-vaccination imaging under a fixed acquisition and reading protocol, so increased detection cannot be separated from increased incidence.
Evidence of incident formation or accelerated growth (coherence)	Where nodules were followed, they regressed under immunosuppression, indicating an inflammatory process [16], or remained stable with neither progression nor regression [8]; in a large screening cohort the high-risk proportion did not rise despite more nodules being detected [11].	No standardized volumetric or longitudinal data demonstrate vaccine-associated new nodule formation or accelerated growth.

No single criterion is currently met by direct, individual-level evidence. Taken together, the table shows that current evidence is insufficient to establish causality, rather than demonstrating the absence of any effect. AI, artificial intelligence; CT, computed tomography; [18F]FDG-PET/CT, [18F]fluorodeoxyglucose positron emission tomography/computed tomography; LDCT, low-dose computed tomography.

**Table 4:** Practical interpretation of pulmonary or thoracic findings detected after COVID-19 vaccination.

Scenario	More Plausible Explanation	Recommended Interpretation
Small incidental CT nodule detected after vaccination	Pre-existing incidental nodule, screening detection or infection-era imaging increase	Do not attribute causality to vaccination on timing alone. Manage by size, density, morphology, growth and patient risk.
[18F]FDG-avid ipsilateral axillary nodes after recent vaccination	Reactive vaccine-associated lymphadenopathy	Document vaccine timing and injection site. Prefer short-interval follow-up or contextual interpretation over immediate upstaging when clinically appropriate.
Pulmonary nodules with fever, uveitis, rash or bilateral hilar/mediastinal nodes	Granulomatous disease, including sarcoidosis-like disease	Evaluate systemic features and alternative causes. Case reports support awareness, not population-level risk inference.
Pulmonary nodules with hemoptysis, sinonasal symptoms, renal findings or PR3-ANCA positivity	ANCA-associated vasculitis, including granulomatosis with polyangiitis	Prompt rheumatology or pulmonary evaluation may be warranted. Do not collapse systemic vasculitis into routine vaccine attribution.
New nodules after COVID-19 infection or during post-COVID follow-up	Residual inflammatory, organizing-pneumonia or fibrotic-like abnormalities	Use the infection timeline, serial imaging and guideline-based risk stratification to distinguish resolution, persistence and growth.

## Data Availability

No new data were created or analyzed in this study. Data sharing is not applicable to this article.

## Supplementary Materials

The following supporting information can be downloaded at: https://www.mdpi.com/article/10.3390/vaccines14090731/s1, Search Strategy Appendix, reporting the databases, search strings, search dates and record-level eligibility decisions for the PubMed and Cochrane Library searches. Table S1: Summary of case reports of pulmonary parenchymal nodular findings after COVID-19 vaccination discussed in this review.

## Abbreviations

AI, artificial intelligence; ANCA, antineutrophil cytoplasmic antibody; COPD, chronic obstructive pulmonary disease; CT, computed tomography; [18F]FDG, [18F]fluorodeoxyglucose; [18F]FDG-PET/CT, [18F]fluorodeoxyglucose positron emission tomography/computed tomography; FVC, forced vital capacity; GPA, granulomatosis with polyangiitis; IPF, idiopathic pulmonary fibrosis; IVW, inverse variance weighted; LDCT, low-dose computed tomography; PR3, proteinase 3; SARS-CoV-2, severe acute respiratory syndrome coronavirus 2; SUVmax, maximum standardized uptake value.
