# Supplementary material for: COVID-19 Vaccination and Pulmonary Nodules: A Narrative Review of Causality, Detection Bias and Thoracic Imaging Pitfalls
Source: Vaccines (Basel). 2026 Aug 25;14(9):731. doi: 10.3390/vaccines14090731 (PMC13611786; doi:10.3390/vaccines14090731)
Supplement: Supplementary file 1 [file vaccines-14-00731-s001.zip › vaccines-4478860-Supplementary Materials — Search Strategy Appendix.pdf]

Supplementary Materials — Search Strategy Appendix

COVID-19 Vaccination and Pulmonary Nodules: A Narrative Review of Causality, Detection Bias and Thoracic Imaging Pitfalls

This appendix reports the databases searched, the exact search strings, the final search date, the screening workflow and the record counts, to support the reproducibility of this narrative review. This was a focused narrative review rather than a systematic review; no PRISMA screening, risk-of-bias scoring or quantitative synthesis was performed.

1. Databases and final search date

Databases: PubMed and the Cochrane Library (Cochrane Database of Systematic Reviews, CDSR, and the Cochrane Central Register of Controlled Trials, CENTRAL). PubMed Central and selected journal platforms were consulted for full-text retrieval of records already identified.

Final search date: 15 July 2026. No date or language restriction was applied.

2. Search concept blocks

Core search strings combined the following concept blocks with the Boolean operator AND across blocks and OR within each block:

- **COVID-19 vaccination:** COVID-19 vaccin*, SARS-CoV-2 vaccin*, mRNA vaccin*, booster (and the MeSH term "COVID-19 vaccines")
- **Pulmonary nodules:** pulmonary nodule*, lung nodule*, micronodule*, solitary pulmonary nodule (and the MeSH terms "Multiple Pulmonary Nodules" and "Solitary Pulmonary Nodule")
- **Lung-disease outcomes:** lung cancer, pneumonia, idiopathic pulmonary fibrosis, pulmonary embolism, pneumothorax, sarcoidosis, asthma, COPD
- **Imaging findings:** [18F]FDG-PET/CT, lymphadenopathy, axillary node, post-COVID CT
- **Detection-bias terms:** artificial intelligence, AI-assisted detection, low-dose CT, screening

3. Exact final query strings

PubMed — primary (core) query [vaccination × pulmonary nodules]:

```
("COVID-19 vaccines"[MeSH] OR "COVID-19 vaccin*"[tiab] OR "SARS-CoV-2 vaccin*"[tiab] OR "mRNA
vaccin*"[tiab] OR booster[tiab]) AND ("pulmonary nodule*"[tiab] OR "lung nodule*"[tiab] OR
micronodule*[tiab] OR "solitary pulmonary nodule"[tiab] OR "Multiple Pulmonary Nodules"[MeSH] OR
"Solitary Pulmonary Nodule"[MeSH])
```

This primary query retrieved 9 records (see Section 4).

PubMed — broad (context) query [vaccination × lung-disease/imaging/detection]:

```
("COVID-19 vaccin*"[tiab] OR "SARS-CoV-2 vaccin*"[tiab] OR "mRNA vaccin*"[tiab] OR
booster[tiab]) AND ("lung cancer"[tiab] OR pneumonia[tiab] OR "idiopathic pulmonary
fibrosis"[tiab] OR "pulmonary embolism"[tiab] OR pneumothorax[tiab] OR sarcoidosis[tiab] OR
asthma[tiab] OR COPD[tiab] OR "FDG-PET"[tiab] OR "PET/CT"[tiab] OR lymphadenopathy[tiab] OR
"axillary node*"[tiab] OR "post-COVID CT"[tiab] OR "artificial intelligence"[tiab] OR "low-dose
CT"[tiab] OR screening[tiab])
```

This broad query was used only to identify background and contextual literature. Because it contains high-frequency general terms (for example, screening, asthma, artificial intelligence), it retrieved a very large record set (approximately 3,000 records) dominated by material unrelated to vaccine–nodule causality; it was therefore screened for relevance and only a small number of contextual references were retained. It was not used to define the direct-evidence set and is not carried into the record-count flow below.

Cochrane Library — CDSR and CENTRAL (search syntax):

```
#1  ("COVID-19 vaccin*" OR "SARS-CoV-2 vaccin*" OR "coronavirus vaccin*" OR "mRNA vaccin*") AND
("pulmonary nodule*" OR "lung nodule*" OR "pulmonary micronodule*" OR "solitary pulmonary
nodule*")
#2  ("COVID-19 vaccin*" OR "SARS-CoV-2 vaccin*" OR "mRNA vaccin*") AND (lymphadenopathy OR
"axillary lymph node*" OR "FDG-PET" OR "PET/CT" OR "thoracic imaging" OR "chest CT")
```

In CDSR, both searches returned 0 records. In CENTRAL, search #1 returned 0 records and search #2 returned 9 records (7 independent trials after removing duplicate registry entries); none evaluated COVID-19 vaccination as a cause of pulmonary parenchymal nodules. Of these, one record (ipsilateral axillary lymphadenopathy after an inactivated vaccine) is cited as contextual nodal evidence.

4. Record counts

Stage	Records
PubMed primary query (vaccination × pulmonary nodules)	9
Cochrane CENTRAL (search #2)	9 (7 independent trials)
Cochrane CDSR	0
Records identified in the primary/CENTRAL searches, before de-duplication	18
Duplicate records removed	0
Records after de-duplication	18
Records excluded after title/abstract and full-text screening	11
Records retained (direct, indirect or contextual evidence)	7
— of which core direct/indirect studies in the evidence tables	4
— of which contextual references	3

Notes on the counts: The 9 PubMed records and the 9 CENTRAL records did not overlap (CENTRAL records are trial-registry entries), so no duplicates were removed between databases. From the 9 PubMed records, four were retained as direct or indirect evidence — Boonsong et al. [16], Chen et al. [9], Shirai et al. [14] and Li et al. [11] — and two were retained as contextual imaging references (increased [18F]FDG avidity after vaccination; axillary adenopathy after vaccination); three were excluded (a cardiothoracic-imaging highlights review, a subacute-thyroiditis case, and a case in which pulmonary nodules were incidental to severe COVID-19 infection rather than vaccination). From CENTRAL, one record (Turan et al. [8], ipsilateral axillary lymphadenopathy after an inactivated vaccine) was retained as contextual nodal evidence. Additional contextual references on nodule detection, AI-assisted reading, low-dose CT screening and pulmonary-nodule management were identified through the broad PubMed query and targeted full-text retrieval and are cited as contextual evidence.

5. Eligibility and screening

Records were eligible when they addressed COVID-19 vaccination together with pulmonary nodules, lung-disease outcomes, thoracic imaging findings, post-COVID CT abnormalities, AI-assisted nodule detection or pulmonary nodule management. Peer-reviewed studies with available abstracts or full texts were prioritized. Case reports were retained only when they addressed vaccination together with nodular or granulomatous lung findings. Records in which pulmonary nodules were incidental to SARS-CoV-2 infection rather than vaccination, and records without a thoracic nodular or relevant imaging outcome, were excluded from the evidence tables.

Title/abstract and full-text screening were performed independently by two authors (J.H. and Y.L.). Disagreements were resolved by discussion and, where necessary, adjudication by the senior author (M.T.).

6. Evidence classification

Eligible studies were classified into three tiers: direct (individual-level vaccination exposure with pulmonary-nodule outcomes), indirect (vaccination with broader lung-disease outcomes or ecological

detection trends) and contextual (vaccine-associated lymphadenopathy imaging, rare immune-mediated case reports, post-COVID CT follow-up, AI-assisted detection studies and pulmonary nodule guidelines). This classification was prespecified because studies of imaging pitfalls can inform differential diagnosis yet cannot, by themselves, establish nodule causation.

7. Limitations of the search

This was a focused search appropriate to a narrative review rather than a systematic review. Formal PRISMA-guided screening, risk-of-bias assessment and quantitative synthesis were not undertaken. The primary databases were PubMed and the Cochrane Library; Embase, Web of Science and systematic grey-literature repositories were not searched, and the search may therefore have missed individual reports. The small number of directly relevant records (nine in the primary PubMed query) is itself consistent with the review's central observation that direct evidence on vaccination and pulmonary nodules is sparse.
