# Supplementary material for: COVID-19 Vaccination and Pulmonary Nodules: A Narrative Review of Causality, Detection Bias and Thoracic Imaging Pitfalls
Source: Vaccines (Basel). 2026 Aug 25;14(9):731. doi: 10.3390/vaccines14090731 (PMC13611786; doi:10.3390/vaccines14090731)
Supplement: Supplementary file 1 [file vaccines-14-00731-s001.zip › vaccines-4478860-supplementary.pdf]

Table S1. Summary of case reports of pulmonary parenchymal nodular findings after COVID-19 vaccination discussed in this review.

Reference	Demographics	Vaccine platform and dose	Interval from vaccination to detection	Radiological findings	Diagnostic work-up and causality assessment	Final diagnosis and outcome
Chen et al., 2025 [9]	34-year-old primigravida, previously healthy (mild intermittent asthma during pregnancy)	mRNA COVID-19 vaccine, four doses, approximately 12 months preconception; no SARS-CoV-2 infection history	Approximately 18-month span from vaccination to detection; micronodules found incidentally on routine 6-month postpartum CT	3–5 randomly distributed pulmonary micronodules (1–3 mm) with no lymphadenopathy; two benign thyroid cystic nodules (largest 0.26 cm, TI-RADS 1); asymptomatic	Serial imaging (CT every 6 months; thyroid ultrasound every 12 months); metastasis, granulomatous disease and classic autoimmune disease excluded; normal thyroid function tests and C-reactive protein. The authors state that a single case cannot establish causality and note recall bias and unmeasured confounders; the report is hypothesis-generating.	Benign pulmonary micronodules with benign thyroid cysts; no pharmacological treatment; neither improvement nor deterioration during follow-up.
Shirai et al., 2023 [14]	63-year-old woman, previously healthy	Pfizer-BioNTech (BNT162b2) mRNA vaccine, first dose	Ear, nose and throat symptoms 3 days after the dose; multiple pulmonary nodules on CT by approximately 22 days; chest radiograph normal 3 months before vaccination	Paranasal-sinus soft-tissue shadows with multiple pulmonary nodules on CT; nasal-mucosal biopsy (not lung) consistent with granulomatosis with polyangiitis	PR3-ANCA positive (259 U/mL; normal <3.5), MPO-ANCA negative; C-reactive protein 14.32 mg/dL; normal renal function and urinalysis. The authors note that a causal relationship is difficult to demonstrate.	PR3-ANCA-positive granulomatosis with polyangiitis; treated with prednisolone 60 mg/day and intravenous cyclophosphamide; follow-up CT showed regression of the lung nodules, consistent with an inflammatory process.
Boonsong et al., 2023 [16]	51-year-old woman, previously healthy, non-smoker; worked as a chef	ChAdOx1-S (recombinant); dose number not specified in the report	Progressive exertional dyspnoea for approximately 2 months after vaccination	Diffuse ground-glass opacities with multiple small centrilobular ground-glass nodules and branched buds on high-resolution CT; no mediastinal lymphadenopathy; mild restrictive ventilatory defect	Hypersensitivity pneumonitis and tuberculosis excluded; antinuclear antibody weakly positive (1:80) with negative myositis-specific antibodies and rheumatoid factor; tissue	Pulmonary talcosis (biopsy-confirmed); vaccination was the temporal trigger that prompted evaluation rather than the cause; supportive care, with resolution of respiratory symptoms on follow-up.

Reference	Demographics	Vaccine platform and dose	Interval from vaccination to detection	Radiological findings	Diagnostic work-up and causality assessment	Final diagnosis and outcome
					biopsy showed foreign-body granuloma with birefringent crystalline particles on polarized microscopy; history of daily cosmetic-talc inhalation approximately 10 years earlier.	

We restricted this table to reports whose principal thoracic finding is a pulmonary parenchymal nodule. Reports whose principal finding is nodal or non-nodular are discussed in the main text (Sections 3.2 and 3.3) rather than listed here, to avoid classifying nodal or ground-glass findings as parenchymal nodules. These include the case of COVID-19 mRNA vaccine–associated uveitis leading to a diagnosis of sarcoidosis, in which the thoracic findings were right-lung ground-glass and granular opacity with hilar and mediastinal lymphadenopathy diagnosed by parotid-gland biopsy rather than a parenchymal nodule [15]; the single-center [18F]FDG-PET/CT series of vaccine-associated axillary lymphadenopathy (75/140 [54%] FDG-avid ipsilateral nodes, mean SUVmax 5.1; Moderna 72% vs. Pfizer-BioNTech 43%) [6]; and the inactivated-vaccine ultrasonography series [8].

ANA, antinuclear antibody; ANCA, antineutrophil cytoplasmic antibody; CT, computed tomography; [18F]FDG-PET/CT, [18F]fluorodeoxyglucose positron emission tomography/computed tomography; GPA, granulomatosis with polyangiitis; MPO, myeloperoxidase; PR3, proteinase 3; SUVmax, maximum standardized uptake value; TI-RADS, Thyroid Imaging Reporting and Data System.
